# Supplementary material for: Asymptomatic hypoglycemia among preterm newborns: A cross-sectional analysis
Source: PLoS One. 2024 Apr 30;19(4):e0301803. doi: 10.1371/journal.pone.0301803 (PMC11060517; doi:10.1371/journal.pone.0301803)
Supplement: S1 Dataset — (PDF) [file pone.0301803.s001.pdf]

Idnumb	Interdate	Time1	Center	Q1Date1	Q2Educatn	Q3Maristat
1	20-Jun-2022	930	TRRH		2	2
2	20-Jun-2022	1,002	TRRH		2	2
3	20-Jun-2022	1,035	TRRH		2	2
4	20-Jun-2022	1,220	TRRH		1	1
5	20-Jun-2022	1,250	TRRH		1	1
6	20-Jun-2022	1,550	TRRH		3	2
7	21-Jun-2022	1,052	TRRH		1	5
8	21-Jun-2022	1,030	TRRH		2	2
9	22-Jun-2022	930	TRRH		2	2
10	22-Jun-2022	1,040	TRRH		2	2
11	22-Jun-2022	1,030	TRRH		2	2
12	22-Jun-2022	1,610	TRRH		1	2
13	22-Jun-2022	2,030	TRRH		3	2
14	22-Jun-2022	2,110	TRRH		2	2
15	22-Jun-2022	2,150	TRRH		2	2
16	22-Jun-2022	2,240	TRRH		2	2
17	23-Jun-2022	1,700	TRRH		3	2
18	23-Jun-2022	1,750	TRRH		2	2
19	23-Jun-2022	1,807	TRRH		3	2
20	23-Jun-2022	1,020	TRRH		3	3
21	24-Jun-2022	1,830	TRRH		2	3
22	24-Jun-2022	1,855	TRRH		2	3
23	24-Jun-2022	1,000	TRRH		2	3
24	23-Jun-2022	1,730	TRRH		3	2
25	26-Jun-2022	1,630	TRRH		3	2
26	26-Jun-2022	1,547	TRRH		3	2
27	26-Jun-2022	930	TRRH		2	1
28	26-Jun-2022	815	TRRH		3	2
29	26-Jun-2022	1,557	TRRH		3	1
30	26-Jun-2022	1,630	TRRH		2	2
31	29-Jun-2022	1,000	TRRH		3	2
32	29-Jun-2022	1,220	TRRH		2	2
33	29-Jun-2022	1,240	TRRH		2	2
34	29-Jun-2022	1,330	TRRH		3	2
35	29-Jun-2022	1,400	TRRH		3	2
36	09-Jul-2022	604	TRRH		2	2
37	09-Jul-2022	1,010	TRRH		4	1
38	09-Jul-2022	1,120	TRRH		4	2
39	04-Jul-2022	1,530	TRRH		3	2
40	01-Jul-2022	1,120	TRRH		2	1
41	01-Jul-2022	1,200	TRRH		2	1
42	01-Jul-2022	1,240	TRRH		1	2
43	01-Jul-2022	1,300	TRRH		4	2
44	05-Jul-2022	1,850	TRRH		2	1
45	05-Jul-2022	1,710	TRRH		2	2
46	05-Jul-2022	1,730	TRRH		2	2
47	04-Jul-2022	1,330	TRRH		2	2
48	05-Jul-2022	1,810	TRRH		2	2
49	15-Jul-2022	1,200	TRRH		1	2
50	15-Jul-2022	1,230	TRRH		1	2
51	16-Jul-2022	1,320	TRRH		3	2
52	16-Jul-2022	1,350	TRRH		3	2
53	16-Jul-2022	1,400	TRRH		3	1
54	16-Jul-2022	1,420	TRRH		3	1
55	16-Jul-2022	1,526	TRRH		1	1

56	16-Jul-2022	1,800	TRRH	4	2
57	16-Jul-2022	1,830	TRRH	4	2
58	19-Jul-2022	1,506	TRRH	2	2
59	19-Jul-2022	1,550	TRRH	2	2
60	19-Jul-2022	1,235	TRRH	2	2
61	19-Jul-2022	1,250	TRRH	2	2
62	22-Jul-2022	1,230	TRRH	3	1
63	22-Jul-2022	1,250	TRRH	3	1
64	22-Jul-2022	1,330	TRRH	2	2
65	22-Jul-2022	1,510	TRRH	2	1
66	22-Jul-2022	1,700	TRRH	2	2
67	22-Jul-2022	1,840	TRRH	2	1
68	23-Jun-2022	900	MRRH	3	2
69	23-Jun-2022	1,010	MRRH	2	1
70	23-Jun-2022	1,610	MRRH	3	2
71	23-Jun-2022	1,846	MRRH	2	2
72	22-Jun-2022	1,640	MRRH	2	2
73	24-Jun-2022	1,430	MRRH	4	2
74	24-Jun-2022	1,010	MRRH	4	2
75	24-Jun-2022	1,235	MRRH	2	2
76	24-Jun-2022	900	MRRH	3	1
77	25-Jun-2022	1,210	MRRH	2	2
78	24-Jun-2022	1,733	MRRH	3	2
79	25-Jun-2022	1,800	MRRH	3	2
80	25-Jun-2022	1,100	MRRH	2	2
81	25-Jun-2022	1,545	MRRH	3	2
82	25-Jun-2022	900	MRRH	4	1
83	26-Jun-2022	815	MRRH	3	2
84	26-Jun-2022	1,910	MRRH	2	2
85	26-Jun-2022	1,201	MRRH	4	2
86	26-Jun-2022	1,231	MRRH	4	2
87	26-Jun-2022	1,310	MRRH	2	2
88	27-Jun-2022	1,210	MRRH	3	2
89	27-Jun-2022	1,540	MRRH	2	2
90	27-Jun-2022	1,620	MRRH	3	2
91	27-Jun-2022	1,720	MRRH	3	2
92	29-Jun-2022	925	MRRH	2	2
93	29-Jun-2022	1,010	MRRH	2	2
94	01-Jul-2022	910	MRRH	3	1
95	01-Jul-2022	1,000	MRRH	3	1
96	02-Jul-2022	750	MRRH	3	1
97	02-Jul-2022	1,900	MRRH	2	2
98	02-Jul-2022	1,930	MRRH	2	2
99	04-Jul-2022	2,000	MRRH	4	2
100	04-Jul-2022	2,100	MRRH	4	2
101	04-Jul-2022	1	MRRH	2	2
102	04-Jul-2022	41	MRRH	2	2
103	05-Jul-2022	1,200	MRRH	4	2
104	05-Jul-2022	1,840	MRRH	2	1
105	05-Jul-2022	1,920	MRRH	2	1
106	06-Jul-2022	710	MRRH	3	2
107	08-Jul-2022	800	MRRH	4	2
108	08-Jul-2022	830	MRRH	4	2
109	07-Jul-2022	1,520	MRRH	3	2
110	08-Jul-2022	900	MRRH	3	1
111	08-Jul-2022	938	MRRH	3	1

112	09-Jul-2022	1,400	MRRH	3	1
113	09-Jul-2022	1,430	MRRH	3	1
114	10-Jul-2022	1,620	MRRH	4	2
115	10-Jul-2022	1,650	MRRH	4	2
116	11-Jul-2022	730	MRRH	3	2
117	12-Jul-2022	910	MRRH	3	2
118	24-Jun-2022	1,030	ARRH	3	2
119	26-Jun-2022	830	ARRH	3	2
120	25-Jun-2022	1,002	ARRH	2	2
121	25-Jun-2022	1,048	ARRH	3	2
122	25-Jun-2022	1,100	ARRH	3	2
123	25-Jun-2022	1,244	ARRH	3	2
124	25-Jun-2022	1,259	ARRH	1	2
125	26-Jun-2022	1,448	ARRH	3	1
126	25-Jun-2022	1,630	ARRH	4	1
127	26-Jun-2022	720	ARRH	3	1
128	26-Jun-2022	815	ARRH	2	2
129	26-Jun-2022	850	ARRH	2	2
130	26-Jun-2022	1,014	ARRH	2	2
131	26-Jun-2022	1,048	ARRH	3	5
132	26-Jun-2022	1,103	ARRH	2	2
133	26-Jun-2022	1,409	ARRH	3	2
134	26-Jun-2022	1,540	ARRH	3	1
135	26-Jun-2022	1,618	ARRH	2	2
136	26-Jun-2022	1,832	ARRH	3	2
137	26-Jun-2022	2,330	ARRH	3	2
138	30-Jun-2022	1,840	ARR	2	2
139	01-Jul-2022	832	ARRH	2	2
140	01-Jul-2022	1,123	ARRH	2	2
141	01-Jul-2022	1,102	ARRH	2	2
142	01-Jul-2022	1,630	ARRH	3	2
143	01-Jul-2022	1,802	ARRH	3	1
144	01-Jul-2022	1,832	ARRH	3	1
145	02-Jul-2022	1,030	ARRH	3	2
146	02-Jul-2022	1,045	ARRH	2	2
147	02-Jul-2022	1,230	ARRH	4	2
148	02-Jul-2022	1,250	ARRH	3	2
149	02-Jul-2022	1,355	ARRH	2	1
150	02-Jul-2022	1,728	ARRH	3	1
151	02-Jul-2022	1,610	ARRH	3	2
152	02-Jul-2022	1,822	ARRH	3	2
153	02-Jul-2022	2,018	ARRH	4	2
154	06-Jul-2022	1,000	ARRH	2	2
155	06-Jul-2022	1,041	ARRH	3	2
156	06-Jul-2022	1,430	ARRH	3	5
157	06-Jul-2022	1,732	ARRH	2	2
158	06-Jul-2022	1,742	ARRH	3	3
159	06-Jul-2022	1,912	ARRH	4	1
160	07-Jul-2022	942	ARRH	4	1
161	07-Jul-2022	1,100	ARRH	2	2
162	07-Jul-2022	1,320	ARRH	2	2
163	07-Jul-2022	1,350	ARRH	3	2
164	07-Jul-2022	1,400	ARRH	3	1
165	07-Jul-2022	1,442	ARRH	3	1
166	07-Jul-2022	1,532	ARRH	2	2
167	07-Jul-2022	1,610	ARRH	3	2

168	10-Jul-2022	1,315	ARRH	3	2
169	10-Jul-2022	1,610	ARRH	3	2
170	10-Jul-2022	630	ARRH	3	2
171	11-Jul-2022	2,012	ARRH	3	2
172	12-Jul-2022	1,440	ARRH	3	1
173	13-Jul-2022	920	ARRH	3	2
174	13-Jul-2022	1,032	ARRH	3	1
175	13-Jul-2022	1,100	ARRH	3	1
176	13-Jul-2022	1,532	ARRH	3	2
177	14-Jul-2022	700	ARRH	3	1
178	15-Jul-2022	930	ARRH	3	2
179	15-Jul-2022	1,000	ARRH	3	2
180	15-Jul-2022	1,500	ARRH	2	2
181	16-Jul-2022	950	ARRH	3	2
182	16-Jul-2022	1,120	ARRH	3	1
183	16-Jul-2022	230	ARRH	2	2
184	16-Jul-2022	432	ARRH	3	2
185	18-Jul-2022	1,000	ARRH	3	2
186	18-Jul-2022	1,040	ARRH	3	2
187	18-Jul-2022	1,320	ARRH	3	2
188	18-Jul-2022	1,500	ARRH	3	1
189	19-Jul-2022	2,040	ARRH	3	2
190	20-Jul-2022	600	ARRH	2	2
191	20-Jul-2022	730	ARRH	3	1
192	20-Jul-2022	1,000	ARRH	4	2
193	20-Jul-2022	1,130	ARRH	4	2
194	20-Jul-2022	1,230	ARRH	4	2
195	20-Jul-2022	2,000	ARRH	4	2
196	21-Jul-2022	803	ARRH	3	1
197	21-Jul-2022	1,000	ARRH	1	2
198	21-Jul-2022	1,700	ARRH	4	2
199	22-Jul-2022	1,002	ARRH	2	2
200	22-Jul-2022	1,030	ARRH	2	2
201	22-Jul-2022	1,050	ARRH	3	2
202	22-Jul-2022	1,350	ARRH	3	1
203	22-Jul-2022	2,027	ARRH	3	2
204	23-Jul-2022	1,110	ARRH	3	2
205	23-Jul-2022	1,132	ARRH	4	2
206	23-Jul-2022	1,440	ARRH	3	1
207	23-Jul-2022	1,847	ARRH	3	1
208	24-Jul-2022	1,040	ARRH	4	2
209	24-Jul-2022	1,345	ARRH	4	2
210	24-Jul-2022	1,400	ARRH	3	2
211	24-Jul-2022	1,530	ARRH	4	2
212	24-Jul-2022	1,930	ARRH	3	2
213	25-Jul-2022	1,542	ARRH	4	2
214	25-Jul-2022	2,200	ARRH	4	2
215	25-Jul-2022	2,230	ARRH	3	2
216	26-Jul-2022	1,630	ARRH	3	2
217	26-Jul-2022	2,200	ARRH	4	2

Q4Employstat	Q5SystBP	Q5DistBP	Q6Preecla	Q7Diabetstat	Q8Dextroinfu	Q9Hemoglo
2	112	70	2	2	2	12.3
2	110	70	2	2	2	12.5
2	113	70	2	2	2	12.3
1	170	110	1	2	2	10.2
1	170	110	1	2	2	10.2
2	110	80	2	2	1	9.5
3	145	113	1	2	1	10.6
2	156	122	1	2	1	9.3
2	162	110	1	2	2	13.0
3	115	65	2	2	2	10.0
2	123	84	2	2	2	10.9
3	100	68	2	2	2	9.8
1	120	70	2	2	2	10.7
1	120	77	2	2	2	12.7
1	120	77	2	2	2	12.7
1	120	77	2	2	2	12.7
3	120	80	2	2	2	11.1
2	123	84	2	2	2	10.9
3	125	70	2	2	2	9.7
3	116	64	2	2	2	12.5
3	118	72	2	2	2	10.8
3	118	72	2	2	2	10.8
1	134	90	1	2	2	10.2
3	132	85	2	2	2	10.5
3	128	82	2	2	2	10.8
2	112	66	2	2	2	11.2
3	108	64	2	2	2	11.2
3	112	66	2	2	2	13.6
1	118	70	2	2	2	10.4
3	114	64	2	2	2	11.0
3	91	44	2	2	2	9.9
2	120	80	2	2	2	11.6
2	120	80	2	2	2	11.6
3	97	65	2	2	2	12.2
3	175	126	1	2	2	9.6
3	120	70	2	2	2	10.7
1	111	61	2	2	2	9.9
1	114	71	2	2	2	12.6
2	118	69	2	2	2	10.9
3	111	72	2	2	2	9.2
3	111	72	2	2	2	9.2
3	113	55	2	2	2	12.0
4	110	76	2	2	2	10.2
1	111	76	2	2	2	11.0
3	104	74	2	2	2	11.0
3	116	78	2	2	2	10.2
2	115	78	2	2	2	10.9
2	117	71	2	2	2	11.0
2	120	76	2	2	2	11.2
2	120	76	2	2	2	11.2
2	120	70	2	2	2	11.1
2	120	70	2	2	2	11.1
3	128	70	2	2	2	10.0
3	125	70	2	2	2	9.9
3	118	60	2	2	2	11.9

3	119	81	2	2	2	12.0
3	119	81	2	2	2	12.0
2	107	62	2	2	2	10.8
2	107	62	2	2	2	10.8
3	202	120	1	2	2	12.2
3	202	120	1	2	2	12.2
4	116	80	2	2	2	11.2
4	116	80	2	2	2	11.2
2	112	56	2	2	2	9.8
2	99	54	2	2	2	11.2
2	117	63	2	2	2	10.8
3	107	63	2	2	2	10.2
3	145	110	1	2	2	13.0
1	135	90	2	2	2	14.0
2	135	95	2	2	2	10.0
2	140	110	1	2	2	14.0
2	122	98	2	2	2	10.7
2	120	80	2	2	2	13.0
4	130	75	2	2	2	11.0
4	120	85	2	2	2	11.0
3	115	95	2	2	2	12.0
2	120	70	2	2	2	13.0
2	137	70	2	2	2	13.0
2	120	90	2	2	2	10.0
2	130	90	2	2	2	12.0
2	125	75	2	2	2	10.0
4	120	70	2	2	2	14.0
2	120	70	2	2	2	12.0
1	120	85	2	2	2	14.0
4	140	110	1	2	2	12.0
4	140	110	1	2	2	12.0
2	116	78	2	2	2	10.3
2	122	70	2	2	2	14.0
4	120	85	2	2	2	13.5
3	112	73	2	2	2	11.2
3	112	73	2	2	2	11.2
2	130	84	2	2	2	9.0
2	130	84	2	2	2	9.0
3	100	67	2	2	2	12.1
3	100	67	2	2	2	12.1
1	112	88	2	2	2	12.0
3	128	76	2	2	2	11.0
3	128	76	2	2	2	11.0
4	128	69	2	2	2	9.0
3	120	60	2	2	2	10.0
2	168	115	1	2	2	14.0
2	168	115	1	2	2	14.0
4	130	85	2	2	2	8.0
1	156	110	1	2	2	11.2
1	156	110	1	2	2	11.2
2	117	70	2	2	2	13.0
4	158	100	1	2	2	11.0
4	158	100	1	2	2	11.0
3	127	78	2	2	2	9.0
4	127	86	2	2	2	12.0
4	127	86	2	2	2	12.0

4	150	100	1	2	2	9.0
4	150	100	1	2	2	9.0
3	120	70	2	2	2	12.0
3	120	70	2	2	2	12.0
2	165	100	1	2	2	11.0
3	110	66	2	2	2	12.0
2	109	79	2	2	2	12.0
3	118	88	2	2	2	11.2
2	98	79	2	2	2	13.0
3	128	82	2	2	2	10.1
3	108	82	2	2	2	11.2
3	90	60	2	2	2	11.6
3	119	88	2	2	2	11.9
1	101	79	2	2	2	14.5
3	122	86	2	2	2	13.5
3	99	72	2	2	2	13.5
2	103	78	2	2	2	14.0
2	112	88	2	2	2	10.7
2	122	98	2	2	2	12.0
3	105	95	2	2	2	11.0
2	109	79	2	2	2	14.0
3	118	88	2	2	2	14.7
1	107	87	2	2	2	14.7
2	109	71	2	2	2	12.1
4	128	82	2	2	2	14.6
4	110	97	2	2	2	12.3
3	123	79	2	2	2	14.7
3	99	73	2	2	2	12.0
4	108	98	2	2	2	13.9
3	114	92	2	2	2	14.2
4	112	88	2	2	2	14.2
3	88	72	2	2	2	6.3
3	104	87	2	1	2	6.4
4	129	84	2	2	2	10.1
2	98	84	2	2	2	11.4
3	106	97	2	2	2	13.7
4	112	87	2	1	2	10.6
1	110	86	2	2	2	7.2
1	105	88	2	2	2	13.9
3	119	77	2	2	2	12.7
4	104	97	2	1	2	11.9
4	103	83	2	2	2	14.0
2	109	71	2	2	2	10.9
3	118	80	2	1	1	13.4
3	139	103	1	1	1	14.6
2	99	77	2	2	2	14.7
3	138	101	1	2	2	11.9
4	114	82	2	2	2	12.0
1	101	79	2	2	2	13.9
3	115	87	2	2	2	16.0
3	111	81	2	2	2	10.8
3	109	77	2	2	2	11.7
1	117	97	2	2	2	13.2
3	105	76	2	2	2	15.0
3	103	83	2	2	2	11.9
3	115	78	2	2	2	14.7

2	108	88	2	2	2	12.7
4	105	92	2	2	2	10.4
4	98	76	2	2	2	10.9
3	101	92	2	2	2	7.6
3	117	95	2	2	2	11.9
4	105	89	2	2	2	10.0
1	103	79	2	2	2	11.0
1	103	83	2	2	2	13.2
2	126	97	2	2	2	16.0
3	98	79	2	2	2	12.4
3	110	80	2	2	2	13.0
3	111	91	2	2	2	12.9
2	120	92	2	2	2	13.0
4	123	91	2	2	2	14.7
1	117	89	2	2	2	12.0
3	110	88	2	2	2	12.3
3	112	96	2	2	2	6.7
2	109	74	2	2	2	13.4
2	109	74	2	2	2	13.4
4	121	89	2	2	2	11.4
1	117	79	2	2	2	14.7
4	107	83	2	2	2	12.0
3	109	79	2	2	2	13.9
1	146	108	1	2	2	12.5
4	122	89	2	2	2	14.0
4	150	100	1	2	2	9.0
4	150	100	1	2	2	9.0
3	125	80	2	2	2	6.0
3	120	74	2	2	2	10.0
1	130	68	2	2	2	8.0
4	120	73	2	2	2	7.0
2	165	118	1	2	2	11.5
2	165	118	1	2	2	11.5
4	139	119	1	2	2	13.9
4	129	83	2	2	2	13.4
3	109	79	2	2	2	11.7
2	118	97	2	2	2	12.7
4	98	79	2	2	2	11.0
3	102	86	2	2	2	14.0
1	119	73	2	2	2	12.3
4	103	93	2	2	2	12.0
4	108	88	2	2	2	12.0
4	99	87	2	2	2	14.7
4	115	84	2	2	2	14.0
4	124	83	2	1	2	12.7
4	118	78	2	2	2	16.0
4	105	89	2	1	2	12.9
2	103	79	2	2	2	13.5
2	107	89	2	2	2	10.9
4	109	91	2	2	2	14.0

Q10Bloodtrans	Q11PreexMSC	Q12Drug	Q12MentDrug	Q12Surg
2		2		2
2		2		2
2		2		2
2		2		2
2		2		2
2		2		2
2		2		2
2		2		2
2		2		2
2		2		2
2		2		2
1		2		2
2		2		2
2		2		2
2		2		2
2		2		2
2		2		2
2		2		2
2		2		2
2		2		2
2		2		2
2		2		2
2		2		2
2		2		2
2		2		2
2		2		2
2		2		2
2		2		2
2		2		2
2		2		2
2		2		2
2		2		2
2		2		2
2		2		2
2		2		2
2		2		2
2		2		2
2		2		2
2		2		2
2		2		2
2		2		2
2		2		2
2		2		2
2		2		2
2		2		2
2		2		2
2		2		2
2		2		2
2	HIV	1	TLE	2
2	HIV	1	TLE	2
2		2		2
2		2		2
2		2		2
2		2		2
2		2		2

2		2		2
2		2		2
2		2		2
2		2		2
2		2		2
2		2		2
2		2		2
2		2		2
2		2		2
2		2		2
2		2		2
2		2		2
2		2		2
2		2		2
2		2		2
2		2		2
2		2		2
2	HIV	1	TLE	2
2	APPENDICITIS	2		1
2		2		2
2	HIV	1	TLE	2
2		2		2
2		2		2
2		2		2
2	HIV	1	TLE	2
2	DIABETES MELLITUS	2		2
2		2		2
2		2		2
2		2		2
2		2		2
2		2		2
2		2		2
2		2		2
2	HIV	2	TLE	2
2		2		2
2		2		2
2	UTI	1	ANTIBIOTICS	2
2	UTI	1	ANTIBIOTICS	2
2		2		2
2		2		2
2	UTI	1	ANTIBIOTICS	2
2		2		2
2		2		2
2	UTI, SEVERE MALARIA MALA	1	ANTIBIOTICS, ANTIMALARIA	2
2	P.U.D, UTI	1	OMEPRAZOLE, ANTIBIOTIC	2
2	HYPERTENSION	2		2
2		2		2
1	ASTHMA	1	SALBUTAMOL INHALER	2
2	HYPERTENSION	1	NIFEDIPINE	2
2	HYPERTENSION	1	NIFEDIPINE	2
2	HIV	1	TLE	2
2		2		2
2		2		2
1	SICKLE CELL ANAEMIA	1	FOLIC ACID	2
2	HYPERTENTION	2		2
2	HYPERTENSION	2		2

2		2		2
2		2		2
2		2		2
2		2		2
2	HYPERTENSION	2	AMILODIPINE	2
2		2		2
2		2		2
2		2		2
2		2		2
2		2		2
2		2		2
2		2		2
2		2		2
2		2		2
2		2		2
2		2		2
2		2		2
2		2		2
2		2		2
2		2		2
2		2		2
2		2		2
2		2		2
2		2		2
2		2		2
2		2		2
2		2		2
2		2		2
2		2		2
2		2		2
2		2		2
1		2		2
1		2		2
2		2		2
2		2		2
2		2		2
2		2		2
1		2		2
2		2		2
2		2		2
2		2		2
2		2		2
2		2		2
2		2		2
2		2		2
2		2		2
2		2		2
2		2		2
2		2		2
2		2		2
2		2		2
2		2		2
2		2		2
2		2		2
2		2		2
2		2		2

2		2		2
2		2		2
2		2		2
1		2		2
2		2		2
2		2		2
2		2		2
2		2		2
2		2		2
2		2		2
2		2		2
2		2		2
2		2		2
2		2		2
2		2		2
2		2		2
1		2		2
2		2		2
2		2		2
2		2		2
2		2		2
2		2		2
2		2		2
2		2		2
2		2		2
1		2		2
1		2		2
1	HIV	1	TLE	2
2		2		2
1	ANAEMIA	1	MOTHER DOES NOT REME	2
1	ANAEMIA, SEVERE MALARIA	1	ARTESUNATE, ALU	2
2	HYPERTENSION	2		2
2	HYPERTENSION	2		2
2		2		2
2		2		2
2		2		2
2		2		2
2		2		2
2		2		2
2		2		2
2		2		2
2		2		2
2		2		2
2		2		2
2		2		2
2		2		2
2		2		2
2		2		2
2		2		2
2		2		2

Q12MentSurg	Q12Radio	Q12MentRadi	Q12Psycho	Q12MentPsych	Q12Others
	2		2		
	2		2		
	2		2		
	2		2		
	2		2		
	2		2		
	2		2		
	2		2		
	2		2		
	2		2		
	2		2		
	2		2		
	2		2		
	2		2		
	2		2		
	2		2		
	2		2		
	2		2		
	2		2		
	2		2		
	2		2		
	2		2		
	2		2		
	2		2		
	2		2		
	2		2		
	2		2		
	2		2		
	2		2		
	2		2		
	2		2		
	2		2		
	2		2		
	2		2		
	2		2		
	2		2		
	2		2		
	2		2		
	2		2		
	2		2		
	2		2		
	2		2		
	2		2		
	2		2		
	2		2		
	2		2		
	2		2		
	2		2		
	2		2		
	2		2		
	2		2		
	2		2		
	2		2		
	2		2		
	2		2		

	2	2	
	2	2	
	2	2	
	2	2	
	2	2	
	2	2	
	2	2	
	2	2	
	2	2	
	2	2	
	2	2	
	2	2	
	2	2	
	2	2	
	2	2	
	2	2	
	2	2	
	2	2	
APPENDICECTO	2	2	
	2	2	
	2	2	
	2	2	
	2	2	
	2	2	
	2	2	
	2	2	LIFE STYLE
	2	2	
	2	2	
	2	2	
	2	2	
	2	2	
	2	2	
	2	2	
	2	2	
	2	2	
	2	2	
	2	2	
	2	2	
	2	2	
	2	2	
	2	2	
	2	2	
	2	2	
	2	2	
	2	2	
	2	2	LIFE STYLE
	2	2	
	2	2	
	2	2	
	2	2	
	2	2	
	2	2	
	2	2	
	2	2	SEVERAL BLOOD TRANSFUS
	2	2	LIFE STYLE, DIET
	2	2	LIFE STYLE, DIET

2	2
2	2
2	2
2	2
2	2
2	2
2	2
2	2
2	2
2	2
2	2
2	2
2	2
2	2
2	2
2	2
2	2
2	2
2	2
2	2
2	2
2	2
2	2
2	2
2	2
2	2
2	2
2	2
2	2
2	2
2	2
2	2
2	2
2	2
2	2
2	2
2	2
2	2
2	2
2	2
2	2
2	2
2	2
2	2
2	2
2	2
2	2
2	2
2	2
2	2
2	2
2	2
2	2
2	2
2	2
2	2

2	2	
2	2	
2	2	
2	2	
2	2	
2	2	
2	2	
2	2	
2	2	
2	2	
2	2	
2	2	
2	2	
2	2	
2	2	
2	2	
2	2	
2	2	
2	2	
2	2	
2	2	
2	2	
2	2	
2	2	
2	2	
2	2	
2	2	
2	2	
2	2	
2	2	
2	2	
2	2	LIFE STYLE
2	2	LIFE STYLE
2	2	
2	2	
2	2	
2	2	
2	2	
2	2	
2	2	
2	2	
2	2	
2	2	
2	2	
2	2	
2	2	
2	2	
2	2	
2	2	
2	2	

S2Q1Parity	S2Q2Ndel	S2Q3NstilBir	S2Q4Nabort	S2Q5Timeactlab	S2Q6DelTim	S2Q7TypPre
1	1	0	0	1,800	231	2
1	1	0	0	1,530	240	2
1	1	0	0	1,750	245	2
0	0	0	0	2,340	610	1
0	0	0	0	2,340	625	1
1	1	0	0	1,700	810	2
1	1	0	2	500	1,250	2
3	3	0	0	1,800	142	2
1	1	0	0	1,230	1,910	2
2	2	0	0	745	2,130	2
1	0	1	0	635	2,320	2
1	1	0	2	730	950	2
2	2	0	0	540	1,305	1
4	4	0	0	650	1,020	1
4	4	0	0	650	1,030	1
4	4	0	0	650	1,110	1
5	4	1	0	1,310	1,949	2
1	1	0	0	50	935	2
2	2	0	0	1,805	1,050	2
2	2	0	0	800	1,250	2
5	5	0	0	715	1,020	1
5	5	0	0	715	1,102	1
3	3	0	0	2,310	240	2
3	2	1	0	1,430	2,210	2
1	1	0	0	440	940	2
1	1	0	0	17	530	2
1	1	0	0	1,600	2,025	2
2	2	0	0	540	1,014	2
2	2	0	0	2,151	213	2
2	2	0	0	400	815	2
1	1	0	0	705	1,120	2
3	2	1	0	1,230	237	1
3	2	1	0	1,230	250	1
1	1	0	0	420	712	2
1	1	0	0	340	530	2
4	4	0	0	1,130	2,123	2
1	1	0	0	2,300	250	2
1	1	0	0	10	330	2
1	1	0	0	30	750	2
2	2	0	0	1,400	1,844	1
2	2	0	0	1,400	1,859	1
3	3	0	0	2,040	517	2
1	1	0	0	1,320	2,010	2
1	1	0	0	730	1,240	2
2	2	0	0	1,345	1,800	2
4	4	0	0	1,305	1,820	2
1	1	0	0	1,050	1,545	2
1	1	0	0	2,300	330	2
3	3	0	0	130	510	1
3	3	0	0	130	550	1
4	4	0	0	1,120	1,708	1
4	4	0	0	1,120	1,713	2
2	1	1	0	2,000	410	2
2	1	1	0	1,110	200	2
2	2	0	0	1,930	2,250	2

3	3	0	0	130	540	1
3	3	0	0	130	527	1
3	3	2	0	140	620	1
3	3	2	0	140	636	1
1	2	0	0	0	412	1
1	2	0	0	0	402	1
1	1	0	0	640	1,500	1
1	1	0	0	640	1,427	1
1	1	0	0	730	1,513	2
3	3	0	0	1,300	1,652	2
1	1	0	0	810	1,826	2
3	3	0	0	1,102	1,928	2
4	3	0	1	1,800	230	2
3	2	0	0	1,800	10	2
2	1	0	0	200	712	2
1	1	0	0	300	1,100	2
3	3	0	0	230	700	2
2	2	0	1	1,336	1,635	2
2	2	0	0	600	1,100	2
4	4	0	0	2,300	100	2
1	1	0	0	1,400	1,930	2
3	3	0	0	2,040	200	2
2	2	0	0	250	1,035	2
5	5	0	0	440	1,109	2
1	1	0	0	840	1,600	2
1	1	0	0	1,000	200	2
1	1	0	1	500	1,000	2
3	3	0	1	1,200	1,950	2
2	2	0	0	1,410	2,024	2
1	1	0	0	800	1,450	1
1	1	0	0	800	1,510	1
5	5	0	0	1,300	1,610	2
5	5	0	1	2,010	46	2
4	3	1	0	1,000	1,615	2
1	1	0	0	10	605	1
1	1	0	0	10	640	1
3	3	0	0	1,900	2,130	1
3	3	0	0	1,900	2,140	1
1	1	0	0	1,910	2,330	1
1	1	0	0	1,910	2,350	1
1	1	0	0	2,000	45	2
2	2	0	0	400	615	1
2	2	0	0	400	625	1
1	1	0	0	800	1,151	2
1	1	0	1	808	1,415	2
1	1	0	0	230	610	1
1	1	0	0	230	621	1
2	2	0	1	0	343	2
4	3	1	0	2,320	941	1
4	3	1	0	2,320	950	1
4	4	0	0	2,040	108	2
2	2	0	0	1,415	2,100	1
2	2	0	0	1,415	2,120	1
2	2	0	1	2,010	2,310	2
2	1	1	0	800	1,110	1
2	1	1	0	800	1,126	1

2	2	0	0	1,300	1,800	1
2	2	0	0	1,300	1,830	1
1	1	0	0	300	940	1
1	1	0	0	300	956	1
2	1	1	0	1,200	1,600	2
1	1	0	0	610	1,123	2
1	1	0	0	2,210	230	2
2	2	0	0	2,300	220	2
2	2	0	0	2,215	115	2
1	1	0	0	2,100	232	2
3	3	0	0	1,915	130	2
2	2	0	0	2,250	350	2
1	1	0	0	220	625	2
1	0	0	1	110	725	2
2	2	0	0	610	1,030	2
1	1	0	0	800	1,336	2
1	1	0	0	1,020	1,535	2
1	1	0	0	1,430	1,935	2
3	3	0	0	1,400	2,010	2
2	2	0	0	2,230	436	2
2	2	0	0	2,310	330	2
2	2	0	0	30	630	2
1	1	0	1	1,600	2,200	2
2	2	0	0	1,330	1,935	2
4	3	1	0	200	620	2
3	3	0	0	1,000	1,312	2
3	3	0	0	620	1,235	2
3	3	0	0	1,830	2,230	2
2	2	0	0	1,500	2,204	2
3	3	0	0	25	442	2
2	2	0	0	17	617	2
1	1	0	0	330	932	2
0	0	0	1	2,230	530	2
1	1	0	0	1,830	2,332	2
3	3	0	0	1,030	1,430	2
2	2	0	0	1,900	0	2
1	1	0	0	15	620	2
0	0	0	0	30	748	2
1	1	0	1	2,100	300	2
2	2	0	0	340	945	2
2	2	0	0	200	710	2
1	1	0	0	630	1,345	2
3	3	0	0	2,330	332	2
2	2	0	0	2,110	220	2
1	1	0	0	30	732	2
4	4	0	0	10	410	2
3	3	0	0	520	1,030	2
2	2	0	0	727	1,142	2
0	0	0	1	1,725	145	2
1	1	0	0	815	1,430	2
6	6	0	0	310	710	2
2	2	0	0	330	730	2
2	2	0	0	122	500	2
3	3	0	0	130	532	2
4	4	0	0	600	910	2
3	3	0	0	420	930	2

1	1	0	0	200	700	2
2	2	0	0	100	700	2
2	2	0	0	517	1,130	2
7	7	0	0	910	1,330	2
0	0	0	0	2,100	522	2
1	1	0	0	1,950	250	2
2	2	0	0	2,122	322	2
0	0	0	0	2,000	300	2
3	3	0	0	2,210	230	2
0	0	0	0	1,800	100	2
0	0	0	0	448	1,148	2
1	1	0	0	600	1,200	2
3	3	0	0	2,120	120	2
1	1	0	0	1,800	130	2
0	0	0	0	2,100	425	2
3	3	0	0	130	530	2
2	2	0	0	120	730	2
1	1	0	0	2,220	350	1
1	1	0	0	2,220	300	1
2	2	0	0	130	640	2
3	2	0	1	2,220	320	2
2	2	0	0	2,230	430	2
3	3	0	0	1,800	2,330	2
2	2	0	0	1,630	2,235	2
3	3	0	0	2,300	300	2
1	1	0	0	1,200	1,615	2
1	1	0	0	1,200	1,608	2
1	0	1	0	630	1,344	2
2	2	0	0	1,900	2,014	2
5	4	1	1	720	1,300	2
2	2	0	1	730	1,000	2
4	3	1	0	600	1,200	1
4	3	1	0	600	1,220	1
3	3	0	0	2,010	310	2
3	2	1	0	2,145	247	2
2	2	0	0	2,240	550	2
2	2	0	0	2,230	430	2
3	3	0	0	2,310	300	2
2	2	0	0	45	745	2
1	1	0	0	210	920	2
3	3	0	0	2,030	330	2
1	1	0	0	2,315	638	2
3	3	0	0	110	720	2
1	1	0	0	206	820	2
2	1	0	1	230	1,032	2
3	3	0	0	2,320	630	2
2	2	0	0	2,100	300	2
0	0	0	0	100	600	2
2	2	0	0	30	630	2
2	2	0	0	2,030	332	2

S3Q1Date	S3Q1Time	S3Q2Sex	S3Q3Moddel	S3Q4Gestag	S3Q5BirtWeig	S3Q6Apgar1
	231	2	1	210	1,100	7
	240	2	1	210	1,200	7
	245	1	1	210	1,500	8
	610	1	1	245	1,360	4
	625	1	1	245	1,300	7
	810	1	1	245	1,500	4
	1,250	1	1	252	1,300	8
	142	1	1	210	900	7
	1,910	1	1	217	1,100	7
	2,130	2	1	238	1,500	8
	2,320	2	1	245	2,200	6
	950	1	1	231	1,300	8
	1,305	1	1	238	1,400	7
	1,020	2	1	217	1,200	8
	1,030	2	1	217	1,400	7
	1,110	1	1	217	1,100	7
	1,949	2	1	245	1,650	5
	935	2	1	231	1,200	9
	1,050	2	1	252	1,400	7
	1,250	2	1	245	1,400	7
	1,020	2	1	238	1,250	7
	1,102	1	1	238	1,300	7
	240	1	3	252	1,500	9
	2,210	1	1	245	2,100	7
	940	1	1	239	2,300	7
	530	2	1	231	1,700	8
	2,025	1	1	245	1,800	8
	1,014	2	1	238	1,400	9
	213	1	1	217	1,800	8
	815	1	1	245	1,200	7
	1,120	2	1	245	1,000	2
	237	1	1	252	1,600	7
	250	1	1	252	1,500	7
	712	1	1	238	1,200	8
	530	1	1	252	900	8
	2,123	2	1	217	1,700	6
	250	2	1	252	2,300	8
	330	1	1	231	1,200	7
	750	2	1	231	1,600	9
	1,844	1	1	245	1,800	8
	1,859	1	1	245	1,900	8
	517	2	1	252	1,500	7
	2,010	1	3	252	2,100	8
	1,240	2	1	238	1,900	7
	1,800	2	1	252	1,600	8
	1,820	1	1	245	1,700	6
	1,545	2	1	234	2,000	8
	330	1	1	210	1,200	7
	510	1	1	236	2,000	8
	550	1	1	236	1,700	8
	1,708	1	3	254	2,100	8
	1,713	2	3	254	1,800	8
	410	2	1	241	2,000	7
	200	1	1	252	2,000	7
	2,250	1	1	227	1,900	7

540	1	1	231	1,300	5
527	1	1	231	1,700	8
620	1	1	245	1,900	8
636	2	1	245	1,900	6
412	2	1	257	1,900	8
402	1	1	257	2,000	8
1,500	2	1	207	2,000	8
1,427	2	1	207	1,500	8
1,513	2	1	238	1,900	6
1,652	1	1	245	1,800	8
1,826	2	1	231	1,200	8
1,928	1	1	224	1,500	8
330	1	1	203	1,300	7
10	1	1	203	1,400	7
712	1	1	224	1,800	8
1,100	1	1	224	1,400	7
700	2	3	252	1,800	7
1,635	1	1	245	1,900	7
1,100	2	1	224	1,700	8
100	2	1	245	1,900	8
1,930	2	1	210	1,200	8
200	2	1	231	1,300	7
1,035	1	1	189	1,100	8
1,109	1	3	252	1,900	9
1,600	2	1	189	1,400	6
200	2	1	231	1,500	8
1,000	1	1	187	1,000	8
1,950	2	1	238	2,200	8
2,024	2	1	245	2,000	8
1,450	2	4	238	1,700	9
1,510	2	3	238	2,000	7
1,610	1	1	196	900	7
46	1	1	224	1,700	8
1,615	1	3	231	2,000	8
605	2	1	217	1,300	6
640	1	1	217	1,300	8
2,130	1	1	231	2,000	6
2,140	2	1	231	1,600	6
2,330	1	1	255	1,900	8
2,350	1	1	255	1,800	8
45	1	1	196	1,200	7
615	1	1	231	2,300	8
625	1	1	231	2,100	6
1,151	1	1	234	2,300	7
1,415	1	3	231	1,200	6
610	2	1	248	1,500	8
621	2	1	248	1,400	8
343	1	1	240	1,900	8
941	1	1	231	1,700	7
950	1	1	231	1,600	7
108	2	1	246	1,800	6
2,100	1	1	238	1,800	6
2,120	2	1	238	1,800	6
2,310	2	1	243	1,800	8
1,110	2	1	238	1,500	8
1,126	1	1	238	1,500	8

1,800	2	1	252	1,800	8
1,830	2	1	252	1,900	8
940	2	1	238	1,700	8
956	1	1	238	1,400	8
1,600	1	1	231	1,500	7
1,123	2	1	245	1,600	8
230	1	1	196	1,600	7
220	2	1	210	2,100	4
115	1	3	210	1,700	8
232	1	1	224	1,100	8
130	2	1	224	1,200	7
350	2	1	238	2,200	4
625	2	1	168	1,100	7
725	1	1	217	1,700	8
1,030	2	1	210	1,000	9
1,336	1	3	238	2,200	8
1,535	1	3	238	2,000	8
1,935	2	3	238	2,200	8
2,010	2	1	210	1,500	8
436	2	3	238	2,000	4
330	1	1	217	1,300	8
630	2	1	231	1,500	9
2,200	1	1	196	1,100	8
1,935	1	1	217	1,400	5
620	1	1	245	1,400	8
1,312	1	3	252	2,100	6
1,235	2	1	210	1,200	8
2,230	1	1	231	1,800	8
2,204	2	1	224	1,200	8
442	1	1	231	1,400	8
617	1	1	203	1,100	7
932	2	3	224	1,100	7
530	2	3	224	1,700	8
2,332	1	1	210	1,200	8
1,430	2	3	245	2,000	8
0	2	1	238	2,100	9
620	2	1	231	2,300	8
748	2	3	224	1,400	6
300	2	1	210	1,500	8
945	2	1	224	1,900	7
710	1	1	203	1,000	8
1,345	1	1	252	1,500	8
332	1	1	224	1,400	6
220	1	1	224	1,200	8
732	2	1	224	1,300	7
410	2	1	252	2,400	8
1,030	1	1	203	1,100	8
1,142	2	1	224	1,200	8
145	1	1	245	2,100	8
1,430	2	1	224	2,100	8
710	1	2	231	1,250	7
730	2	1	210	1,600	8
500	2	1	224	1,400	8
532	2	1	210	1,500	8
910	2	1	210	1,100	8
930	2	1	210	1,200	8

700	2	1	210	1,100	6
700	2	1	210	1,100	8
1,130	2	1	217	1,100	8
1,330	2	1	196	1,200	6
522	2	1	224	1,900	8
250	1	1	224	2,100	7
322	2	1	224	1,300	8
300	1	1	210	1,700	8
230	2	1	210	1,100	8
100	2	1	203	1,000	8
1,148	1	1	210	1,600	8
1,200	1	1	203	1,100	8
120	2	1	175	800	6
130	1	1	231	1,700	8
425	1	1	224	2,000	6
530	1	1	210	1,600	8
730	2	3	210	1,200	7
350	1	3	231	1,400	7
300	2	3	231	1,700	4
640	2	1	182	1,200	6
320	1	1	182	1,000	8
430	1	1	210	1,300	8
2,330	2	1	217	2,100	8
2,235	2	3	217	2,200	8
300	2	1	196	1,200	8
1,615	2	1	233	1,300	7
1,608	1	1	233	1,400	8
1,344	1	1	231	900	6
2,014	1	1	252	2,000	8
1,300	1	3	238	2,000	7
1,000	1	1	252	2,000	7
1,200	2	1	257	1,300	6
1,220	2	1	257	1,200	7
310	2	1	203	1,000	7
247	2	1	217	1,300	9
550	2	3	210	1,400	7
430	2	2	217	1,700	9
300	2	1	224	2,000	8
745	2	1	203	1,900	8
920	2	1	217	1,200	8
330	2	1	210	1,300	8
638	1	1	210	1,300	8
720	2	3	210	1,500	9
820	1	3	196	1,000	8
1,032	2	3	189	1,200	7
630	2	1	203	1,300	9
300	2	3	210	1,200	7
600	2	3	210	1,500	8
630	2	1	210	1,200	8
332	1	3	196	1,000	8

S3Q6Apgar5	S3Q7Nutrstat	S3Q8BabNic	S3Q9Premat	S3Q9Respdistr	S3Q9Birthasph	S3Q9Mecoas
9	2	1	1	1	2	2
8	2	1	1	2	2	2
9	2	1	1	2	2	2
6	1	1	1	1	1	2
8	1	1	1	1	2	2
6	2	1	1	2	1	2
9	1	2	2	2	2	2
8	2	1	1	1	2	2
9	1	1	1	1	2	2
9	2	2	2	2	2	2
8	2	1	1	1	2	2
9	2	1	1	2	2	2
10	1	2	2	2	2	2
10	2	1	1	1	2	2
10	2	1	1	2	2	2
10	2	1	1	2	2	2
8	2	1	1	1	2	2
10	1	1	1	2	2	2
10	1	2	2	2	2	2
10	1	2	2	2	2	2
10	1	2	2	2	2	2
10	1	2	2	2	2	2
10	1	2	2	2	2	2
10	2	2	2	2	2	2
9	2	2	2	2	2	2
10	2	2	2	2	2	2
10	2	2	2	2	2	2
10	1	2	2	2	2	2
10	2	2	2	2	2	2
9	1	2	2	2	2	2
4	1	1	1	2	1	2
8	1	2	2	2	2	2
8	1	2	2	2	2	2
10	1	1	1	1	2	2
10	1	1	1	2	2	2
9	2	1	1	1	2	2
10	2	1	1	2	2	2
10	2	2	2	2	2	2
10	2	2	2	2	2	2
10	2	2	2	2	2	2
10	2	2	2	2	2	2
9	1	1	1	2	2	2
10	2	2	2	2	2	2
10	2	1	1	2	2	2
10	1	1	1	2	2	2
8	2	1	1	2	2	1
10	2	1	1	2	2	2
10	2	1	1	2	2	2
10	2	1	1	2	2	2
10	2	1	1	2	2	2
10	2	2	1	2	2	2
10	1	2	1	2	2	2
9	2	2	1	1	2	2
9	2	2	1	2	2	2
9	2	2	1	2	2	2

6	2	1	1	2	1	2
10	2	1	1	2	2	2
10	2	2	1	2	2	2
8	2	2	1	2	2	2
10	1	2	1	2	2	2
10	1	2	1	2	2	2
10	3	2	1	2	2	2
10	2	2	1	2	2	2
8	2	1	1	1	2	2
10	2	2	1	2	2	2
10	1	2	1	2	2	2
10	2	2	1	2	2	2
10	2	1	1	1	2	2
10	2	1	1	1	2	2
10	2	1	1	1	2	2
10	2	1	1	1	2	2
10	2	1	1	2	2	2
10	2	1	1	1	2	2
10	2	1	1	2	2	2
10	2	1	1	2	2	2
10	2	1	1	1	2	2
9	2	1	1	2	2	2
10	2	1	1	2	2	2
10	2	1	1	2	2	2
8	2	1	1	2	2	2
10	2	1	1	2	2	2
10	2	1	1	2	2	2
10	2	1	1	2	2	2
10	2	1	1	1	2	2
10	2	1	1	1	2	2
8	2	1	1	1	2	2
9	2	1	1	1	2	2
10	2	1	1	2	2	2
10	2	1	1	2	2	2
8	2	1	1	1	2	2
10	2	1	1	1	2	2
8	2	1	1	2	2	2
8	2	1	1	2	2	2
10	2	1	1	1	2	2
10	2	1	1	1	2	2
10	2	1	1	2	2	2
10	2	1	1	2	2	2
8	2	1	1	2	2	2
10	2	1	1	2	2	2
8	1	1	1	1	2	2
10	1	1	1	1	2	2
10	1	1	1	1	2	2
10	2	1	1	2	2	2
10	2	1	1	2	2	2
10	2	1	1	2	2	2
8	2	1	1	1	2	2
8	2	1	1	2	2	2
8	2	1	1	2	2	2
10	2	1	1	1	2	2
10	2	1	1	1	2	2
10	2	1	1	1	2	2

10	2	1	1	2	2	2
10	2	1	1	2	2	2
10	2	1	1	2	2	2
10	1	1	1	2	2	2
10	2	1	1	2	2	2
10	2	1	1	2	2	2
10	3	1	1	2	2	2
6	3	1	1	2	1	2
10	3	1	1	2	2	2
10	1	1	1	2	2	2
9	2	1	1	1	2	2
6	2	1	1	2	1	2
8	2	1	1	2	2	2
10	2	1	1	2	2	2
10	2	1	1	2	2	2
10	2	1	1	2	2	2
10	2	1	1	2	2	2
10	2	1	1	2	2	2
10	2	1	1	2	2	2
6	2	1	1	2	1	2
10	2	1	1	1	2	2
10	2	1	1	2	2	2
10	2	1	1	1	2	2
7	2	1	1	2	1	2
10	1	1	1	2	2	2
8	2	1	1	2	2	2
10	2	1	1	2	2	2
10	2	1	1	2	2	2
10	2	1	1	2	2	2
10	2	1	1	2	2	2
9	2	1	1	1	1	2
9	1	1	1	2	2	2
9	2	1	1	2	2	2
10	2	1	1	2	2	2
10	2	1	1	2	2	2
10	2	1	1	2	2	2
10	2	1	1	2	2	2
10	2	1	1	2	2	2
10	2	1	1	2	2	2
9	2	1	1	2	2	2
10	2	1	1	2	2	2
10	1	1	1	2	2	2
7	2	1	1	2	1	2
10	2	1	1	2	2	2
8	2	1	1	2	2	1
10	2	1	1	2	2	2
10	2	1	1	2	2	2
10	2	1	1	2	2	2
10	2	1	1	1	2	2
10	3	1	1	2	2	2
9	2	1	1	1	2	2
10	2	1	1	2	2	2
10	2	1	1	2	2	2
9	2	1	1	2	2	2
10	2	1	1	2	2	2
10	2	1	1	2	2	2

9	2	1	1	1	2	2
10	2	1	1	2	2	2
10	2	1	1	2	2	2
8	2	1	1	2	2	2
9	2	1	1	2	2	2
10	3	1	1	2	2	2
10	2	1	1	2	2	2
10	2	1	1	2	2	2
10	2	1	1	2	2	2
10	2	1	1	2	2	2
10	2	1	1	2	2	2
10	2	1	1	2	2	2
7	2	1	1	2	1	2
10	2	1	1	2	2	2
7	2	1	1	2	1	2
10	2	1	1	2	2	2
9	2	1	1	2	2	2
10	2	1	1	2	2	2
6	2	1	1	2	1	2
8	2	1	1	2	2	2
10	2	1	1	2	2	2
10	2	1	1	2	2	2
10	3	1	1	2	2	2
10	3	1	1	2	2	2
10	2	1	1	2	2	2
10	1	1	1	1	2	2
10	2	1	1	1	2	2
9	1	1	1	1	2	2
10	2	1	1	2	2	2
9	2	1	1	2	2	2
10	2	1	1	2	2	2
8	1	1	1	2	2	2
8	1	1	1	2	2	2
9	2	1	1	1	2	2
10	2	1	1	2	2	2
9	2	1	1	2	2	2
10	2	1	1	2	2	2
10	2	1	1	2	2	2
10	3	1	1	2	2	2
10	2	1	1	2	2	2
10	2	1	1	2	2	2
10	2	1	1	2	2	2
10	2	1	1	2	2	2
10	2	1	1	2	2	2
9	2	1	1	2	2	2
10	2	1	1	2	2	2
9	2	1	1	1	2	2
10	2	1	1	2	2	2
10	2	1	1	2	2	2
10	2	1	1	2	2	2

S3Q9Neonse	S3Q9Neonjaun	S3Q9Others	S3Q10Bloodsu	S3Q11Babykanga	S3Q12Babyradinc
2	2		4.2	2	1
2	2		6.2	2	1
2	2		5.0	2	1
2	2		2.2	2	1
2	2		1.8	2	1
2	2		2.6	2	1
2	2		2.1	2	1
2	2		2.4	2	1
2	2		2.3	2	1
2	2		2.7	1	2
2	2		2.1	2	1
2	2		2.8	2	1
2	2		4.1	1	2
2	2		2.1	2	1
2	2		2.6	2	1
2	2		2.8	2	1
2	2		3.2	2	2
2	2		2.1	2	1
2	2		2.8	2	1
2	2		2.9	2	1
2	2		3.2	1	2
2	2		2.5	1	2
2	2		2.3	1	2
2	2		3.6	2	1
2	2		2.6	1	2
2	2		3.2	2	1
2	2		2.8	2	1
2	2		3.4	2	2
2	2		3.6	2	1
2	2		2.1	1	2
2	2		2.1	2	1
2	2		2.0	2	1
2	2		2.4	2	1
2	2		3.6	1	2
2	2		5.4	2	1
2	2		2.4	2	1
2	2	NEC	2.4	2	1
2	2		2.2	2	1
2	2		1.6	2	1
2	2		3.1	2	1
2	2		3.2	2	1
2	2		2.9	2	1
2	2		4.6	2	1
2	2	NEC	3.3	2	1
1	2		2.4	2	1
2	2		7.4	2	1
2	2		6.2	2	1
2	2	NEC	5.2	2	1
2	2	EXPOSED BA	2.6	2	1
2	2	EXPOSED BA	2.4	2	1
2	2		3.2	2	1
2	2		2.5	2	1
2	2		3.3	2	1
2	2		2.0	2	1
2	2		11.2	2	1

2	2		2.0	2	1
2	2	NEC	2.4	2	1
2	2		2.6	2	1
2	2		2.4	2	1
2	2		1.8	2	1
2	2		2.6	2	1
2	2		2.3	2	1
2	2		1.9	2	1
2	2		2.6	2	1
2	2		3.3	2	1
2	2		2.4	2	1
2	2		3.0	2	1
2	2		3.4	2	1
2	2		1.7	2	1
2	2		2.2	2	1
2	2		2.3	2	1
2	2		2.8	2	1
2	2	EXPOSED BA	2.6	2	1
1	2		2.0	2	1
1	2		4.0	2	1
1	2		4.7	2	1
2	2		3.7	2	1
2	2		2.4	2	1
1	2		5.1	2	1
2	2		1.4	2	1
2	2		5.3	2	1
2	2		1.9	2	1
2	2		2.4	2	1
2	2		3.1	2	1
2	2		3.2	2	1
2	2		2.8	2	1
2	2		1.5	2	1
1	2		2.3	2	1
1	2	EXPOSED BA	2.3	2	1
2	2		1.2	2	1
2	2		0.7	2	1
1	2		5.5	2	1
1	2		3.2	2	1
2	2		0.6	2	1
2	2		2.1	2	1
1	2		2.8	2	1
2	2		4.3	2	1
2	2		3.8	2	1
2	2	HYPOTHERM	1.8	2	1
2	2		0.6	2	1
2	2		2.0	2	1
2	2		2.4	2	1
2	2		2.3	2	1
2	2		5.8	2	1
2	2		1.1	2	1
2	2		4.2	2	1
2	2		4.6	2	1
2	2		2.8	2	1
2	2		3.8	2	1
2	2		3.4	2	1
2	2		4.8	2	1

2	2	2.2	2	1
2	2	2.6	2	1
2	2	2.7	2	1
2	2	2.3	2	1
2	2	2.2	2	1
2	2	0.7	2	1
2	2	3.1	2	1
2	2	1.6	2	1
2	2	1.6	2	1
2	2	1.7	2	1
2	2	3.2	2	1
2	2	2.6	2	1
2	2	3.9	2	1
2	2	2.7	2	1
2	2	1.3	2	1
2	2	1.1	2	1
2	2	2.6	2	1
2	2	1.3	2	1
2	2	1.8	2	1
2	2	1.6	2	1
2	2	2.5	2	1
2	2	2.9	2	1
2	2	1.6	2	1
2	2	2.4	2	1
2	2	2.6	2	1
2	2	0.7	2	1
2	2	2.8	2	1
2	2	4.2	2	1
2	2	4.5	2	1
2	2	4.6	2	1
2	2	5.3	2	1
2	2	3.7	2	1
2	2	0.9	2	1
2	2	5.3	2	1
2	2	2.3	2	1
2	2	3.7	2	1
2	2	1.2	2	1
2	2	3.2	2	1
2	2	4.5	2	1
2	2	4.2	2	1
2	2	0.6	2	1
2	2	5.0	2	1
2	2	2.3	2	1
2	2	10.0	2	1
2	2	2.0	2	1
2	2	5.1	2	1
2	2	1.7	2	1
2	2	4.3	2	1
2	2	3.7	2	1
2	2	4.7	2	1
2	2	0.4	2	1
2	2	4.0	2	1
2	2	3.7	2	1
2	2	4.3	2	1
2	2	4.5	2	1
2	2	2.7	2	1

2	2		0.5	2	1
2	2		5.0	2	1
2	2		2.3	2	1
2	2		4.3	2	1
2	2		4.5	2	1
2	2		4.2	2	1
2	2		3.9	2	1
2	2		3.9	2	1
2	2		7.0	2	1
2	2		4.3	2	1
2	2		3.2	2	1
2	2		5.0	2	1
2	2		1.3	2	1
2	2		1.7	2	1
2	2		2.2	2	1
2	2		4.1	2	1
2	2		4.2	2	1
2	2		1.4	2	1
2	2		2.6	2	1
2	2		2.2	2	1
2	2		0.7	2	1
2	2		3.2	2	1
2	2		4.2	2	1
2	2		2.9	2	1
2	2		2.3	2	1
2	2		2.0	2	1
2	2		1.9	2	1
2	2	EXPOSED BA	1.8	2	1
1	2		2.5	2	1
2	2		0.4	2	1
2	2	HYPOTHERM	2.3	2	1
1	2		1.3	2	1
1	2		2.2	2	1
2	2		1.7	2	1
2	2		5.7	2	1
2	2		4.7	2	1
2	2		12.0	2	1
2	2		5.0	2	1
2	2		4.2	2	1
2	2		1.7	2	1
2	2		4.0	2	1
2	2		5.0	2	1
2	2		2.0	2	1
2	2		2.0	2	1
2	2		2.6	2	1
2	2		3.2	2	1
2	2		0.5	2	1
2	2		2.0	2	1
2	2		2.0	2	1
2	2		4.7	2	1

S3Q13Babybreast	S3Q14HealthCadre	Interview time	Time of onset of a
2	7	930	1,800
2	7	1,002	1,530
2	7	1,035	1,750
2	4	1,220	2,340
2	4	1,250	2,340
2	7	1,550	1,700
2	7	1,052	500
2	7	1,030	1,800
2	7	930	1,230
1	7	1,040	745
2	7	1,030	635
2	7	1,610	730
1	7	2,030	540
2	7	2,110	650
2	7	2,150	650
2	7	2,240	650
2	4	1,700	1,310
2	7	1,750	50
2	7	1,807	1,805
2	7	1,020	800
1	7	1,830	715
1	7	1,855	715
1	4	1,000	2,310
1	7	1,730	1,430
2	7	1,630	440
1	7	1,547	17
1	4	930	1,600
2	7	815	540
1	7	1,557	2,151
2	7	1,630	400
2	7	1,000	705
2	7	1,220	1,230
2	7	1,240	1,230
1	7	1,330	420
2	7	1,400	340
2	7	604	1,130
2	7	1,010	2,300
2	7	1,120	10
2	7	1,530	30
2	7	1,120	1,400
2	7	1,200	1,400
1	7	1,240	2,040
2	4	1,300	1,320
2	7	1,850	730
2	7	1,710	1,345
2	7	1,730	1,305
2	7	1,330	1,050
2	7	1,810	2,300
2	7	1,200	130
2	7	1,230	130
2	4	1,320	1,120
2	4	1,350	1,120
1	7	1,400	2,000
2	7	1,420	1,110
1	7	1,526	1,930

2	4	1,800	130
2	4	1,830	130
2	7	1,506	140
2	7	1,550	140
2	7	1,235	0
2	7	1,250	0
2	7	1,230	640
2	7	1,250	640
2	7	1,330	730
2	7	1,510	1,300
2	7	1,700	810
2	7	1,840	1,102
2	7	900	1,800
2	7	1,010	1,800
2	4	1,610	200
2	7	1,846	300
2	4	1,640	230
2	7	1,430	1,336
2	5	1,010	600
2	7	1,235	2,300
2	4	900	1,400
2	7	1,210	2,040
2	7	1,733	250
2	7	1,800	440
2	4	1,100	840
2	7	1,545	1,000
2	7	900	500
2	7	815	1,200
2	7	1,910	1,410
2	1	1,201	800
2	1	1,231	800
2	7	1,310	1,300
2	7	1,210	2,010
2	7	1,540	1,000
2	7	1,620	10
2	7	1,720	10
2	7	925	1,900
2	7	1,010	1,900
2	7	910	1,910
2	7	1,000	1,910
2	4	750	2,000
2	7	1,900	400
2	7	1,930	400
2	7	2,000	800
2	7	2,100	808
2	7	1	230
2	7	41	230
2	4	1,200	0
2	7	1,840	2,320
2	7	1,920	2,320
2	7	710	2,040
2	7	800	1,415
2	7	830	1,415
2	7	1,520	2,010
2	7	900	800
2	7	938	800

2	7	1,400	1,300
2	7	1,430	1,300
2	7	1,620	300
2	7	1,650	300
2	7	730	1,200
2	7	910	610
2	7	1,030	2,210
2	7	830	2,300
2	4	1,002	2,215
2	7	1,048	2,100
1	7	1,100	1,915
2	7	1,244	2,250
2	6	1,259	220
2	7	1,448	110
2	7	1,630	610
2	4	720	800
2	4	815	1,020
2	4	850	1,430
2	6	1,014	1,400
2	4	1,048	2,230
2	7	1,103	2,310
1	4	1,409	30
2	7	1,540	1,600
2	7	1,618	1,330
2	7	1,832	200
2	4	2,330	1,000
1	7	1,840	620
1	7	832	1,830
1	7	1,123	1,500
1	7	1,102	25
1	5	1,630	17
1	4	1,802	330
2	4	1,832	2,230
2	7	1,030	1,830
2	4	1,045	1,030
1	7	1,230	1,900
2	5	1,250	15
1	5	1,355	30
1	7	1,728	2,100
1	7	1,610	340
2	6	1,822	200
2	7	2,018	630
2	7	1,000	2,330
1	7	1,041	2,110
2	4	1,430	30
1	5	1,732	10
2	6	1,742	520
1	7	1,912	727
1	7	942	1,725
1	7	1,100	815
2	4	1,320	310
1	7	1,350	330
1	7	1,400	122
1	7	1,442	130
1	7	1,532	600
1	7	1,610	420

2	7	1,315	200
2	7	1,610	100
2	7	630	517
1	7	2,012	910
1	7	1,440	2,100
1	7	920	1,950
1	7	1,032	2,122
1	7	1,100	2,000
1	7	1,532	2,210
1	7	700	1,800
1	7	930	448
1	7	1,000	600
2	7	1,500	2,120
2	7	950	1,800
2	7	1,120	2,100
1	7	230	130
2	2	432	120
2	4	1,000	2,220
2	4	1,040	2,220
2	7	1,320	130
2	7	1,500	2,220
1	7	2,040	2,230
1	7	600	1,800
2	4	730	1,630
2	7	1,000	2,300
2	7	1,130	1,200
2	7	1,230	1,200
2	7	2,000	630
2	7	803	1,900
2	1	1,000	720
2	7	1,700	730
2	6	1,002	600
2	6	1,030	600
2	7	1,050	2,010
1	7	1,350	2,145
2	4	2,027	2,240
1	6	1,110	2,230
1	7	1,132	2,310
1	5	1,440	45
2	7	1,847	210
1	7	1,040	2,030
1	7	1,345	2,315
2	4	1,400	110
2	4	1,530	206
2	4	1,930	230
1	7	1,542	2,320
2	4	2,200	2,100
2	4	2,230	100
2	7	1,630	30
2	4	2,200	2,030

active labour	Delivery time
	231
	240
	245
	610
	625
	810
	1,250
	142
	1,910
	2,130
	2,320
	950
	1,305
	1,020
	1,030
	1,110
	1,949
	935
	1,050
	1,250
	1,020
	1,102
	240
	2,210
	940
	530
	2,025
	1,014
	213
	815
	1,120
	237
	250
	712
	530
	2,123
	250
	330
	750
	1,844
	1,859
	517
	2,010
	1,240
	1,800
	1,820
	1,545
	330
	510
	550
	1,708
	1,713
	410
	200
	2,250

540
527
620
636
412
402
1,500
1,427
1,513
1,652
1,826
1,928
230
10
712
1,100
700
1,635
1,100
100
1,930
200
1,035
1,109
1,600
200
1,000
1,950
2,024
1,450
1,510
1,610
46
1,615
605
640
2,130
2,140
2,330
2,350
45
615
625
1,151
1,415
610
621
343
941
950
108
2,100
2,120
2,310
1,110
1,126

1,800
1,830
940
956
1,600
1,123
230
220
115
232
130
350
625
725
1,030
1,336
1,535
1,935
2,010
436
330
630
2,200
1,935
620
1,312
1,235
2,230
2,204
442
617
932
530
2,332
1,430
0
620
748
300
945
710
1,345
332
220
732
410
1,030
1,142
145
1,430
710
730
500
532
910
930

700
700
1,130
1,330
522
250
322
300
230
100
1,148
1,200
120
130
425
530
730
350
300
640
320
430
2,330
2,235
300
1,615
1,608
1,344
2,014
1,300
1,000
1,200
1,220
310
247
550
430
300
745
920
330
638
720
820
1,032
630
300
600
630
332
