# Supplementary material for: Asymptomatic hypoglycemia among preterm newborns: A cross-sectional analysis
Source: PLoS One. 2024 Apr 30;19(4):e0301803. doi: 10.1371/journal.pone.0301803 (PMC11060517; doi:10.1371/journal.pone.0301803)
Supplement: Attachment — Submitted filename: Re hypoglycemia study.pdf [file pone.0301803.s002.pdf]

Re: Asymptomatic hypoglycemia among preterm newborns: a cross-sectional analysis.

Thanks for allowing me to review this work. Find below my comments

Short title:

“neonatal hypoglycemia” changes to hypoglycemia in preterm newborns.

Keywords:

“Dar es Salaam” delete, replace with preterm

Abstract

‘There is a growing body of evidence that links the causes of early neonatal mortality to neonatal hypoglycemia in Tanzania’ Replace with another statement, this is a well-established fact globally.

“Written informed consent was sought from each mother prior to inclusion of their babies into the study” delete

“Breastfeeding within 1st hour post-delivery was the only factor significantly associated with neonatal hypoglycemia (OR; 0.123, 95%-CI; 0.052-0.287).” Kindly rephrase to avoid ambiguity. Your odds ratio is less than.

“Nearly half of all asymptomatic newborns were in hypoglycemic range” You can not conclude with findings not in the results section, this is appearing out of no where

“Preterm neonates maybe the risky group worth considering for continuous glycemic screening at hospital protocols” Your abstract failed to support this statement and thus, you can not make this recommendation

Introduction

“They consider their argument on possible evolutionary basis for its occurrence within the first 24-hours of extra-uterine life. They justified their thinking on the nosology to be based on symptoms and signs (clinical aspects),” replace the word “they” and avoid third person pronoun in a scientific writing

“Some studies have shown perinatal hypoxia, small for gestational age and maternal hypertension to be factors associated with hypoglycemia in preterm newborns (4, 5) However, the exact mechanisms behind the observations are yet to be identified by neonatologists/endocrinologists to date. Yet still, it is not clear whether the suggested risks are generalized or specific to a certain neonatal cohort alone” move up to the paragraph before..

“This study gives findings from a cross-sectional sample of preterm neonates who presented with asymptomatic hypoglycemia in typical neonatal set ups” delete

Methods:

“Otherwise, Tanzania is a nation state situated on the East African coast. It is a former German colony, that accidentally fell under British protectorate after the World War II, up to her political independence. Most of her existing infrastructure in health, especially the study settings are post-independence initiatives on health. Tanzania is among the fastest growing nation states in Africa. She is rich in demographic sects, as evidenced by different population sub-structure ranging from

Bantus to Afro-Asiatic population groups, especially in Dar es Salaam." Delete, this is a manuscript and not a dissertation or thesis.

"Thus, the study population was considered to be representative of African demographic sects for all practical and clinical purposes" This is incorrect, kindly delete

"Nutritional status was assessed using growth charts" Mentioned the growth chart name

"Random (venous) blood glucose of the preterm newborns screened using Glucoplus blood glucose monitors (Glucoplus™ Inc. 2004, Canada)" has this been validated among the neonates?

"Data was triple entered under" who do you mean by triple entered? Why?

"(hypoglycemia – coded as 1- if random glucose value was ≥ 2.6 mmol/L and 0 – if random glycaemia was < 2.6 mmol/L)." kindly cross-check, your desired outcome is hypoglycemia and should have been coded 1.

Results:

Apgar score, and weight are appearing here for the first time, how were they assessed and it should be part of the methods. Also, birth asphyxia, respiratory distress, MAS

"TABLE 2: multivariate analysis" it should be bivariate or multivariable binary logistic regression

No mention of how many has hypoglycemia

Discussion

"In this study, we found almost half of all preterm babies to have been in quantitative hypoglycemic ran." This is not mentioned in your results

"The current findings are comparable to others in similar settings (7-10) For instance, Sultan and his colleagues found out 73% of all babies born prematurely in South-Eastern Tanzania to had been in hypoglycemic range" This statement contradict your earlier assertion that no study has been done in Tanzania (At present, there is no evidence from available/retrievable published databases of any findings on factors associated with neonatal hypoglycemia among preterm babies in Tanzania).

"To
what extent do these findings translate to prevalent double burden of morbidity and mortality indices among under-five year population in Tanzania and beyond, is still a matter of speculation (25, 26). However, the fact that there is solid evidence for under-fives population to be in ill-state in Tanzania (22) calls for urgent considerations for more analytical, possibly longitudinal designs in ascertaining the long-term effects ofasymptomatic hypoglycemia in newborns. Besides, these findings call for urgent and reliable estimates of morbidity and mortality statistics of special groups in the population pyramid. At present, there are potential indications that shows certain segments of the Tanzania's population are left out in most reported morbidity and mortality statistics (25, 26). There are potential clues that chronic ill-states in childhood may result to miserable later life years both in Africa and elsewhere (23, 24). As we are advancing medical sciences on ageing cascades, even among humans (19, 24), we wish to consider that as a treasure hunt at least for now." Delete, irrelevant.
