# Supplementary material for: Asymptomatic hypoglycemia among preterm newborns: A cross-sectional analysis
Source: PLoS One. 2024 Apr 30;19(4):e0301803. doi: 10.1371/journal.pone.0301803 (PMC11060517; doi:10.1371/journal.pone.0301803)
Supplement: Attachment — Submitted filename: Hypoglycemia in Preterms.docx [file pone.0301803.s003.docx]

6^th^ Nov 2023

Asymptomatic hypoglycemia among preterm newborns: a cross-sectional analysis.

By : Kelvin Melkizedeck Leshabari, MD, MSc

1. What was definition of Hypoglycemia in Preterm? Consensus on Definition is still unclear in children and newborns. Please discuss it in background and even discussion.
2. So, what was asymptomatic versus symptomatic hypoglycemia? How would u gauge asymptomatic features in a preterm?
3. What was definition of preterm? 37weeks or less. How did you assess Prematurity using either LNMP or Ballard score or both?
4. So which symptoms did you use to exclude symptomatic hypoglycemia in your eligibility criteria?
5. Did you include children who had received IV dextrose infusion if so, how many hours since the infusion?
6. Are the characteristics of the children admitted at these 3 hospitals the same? are they all referral hospitals and not tertiary?
7. Did you find that children from a particular hospital were more likely to get Hypo?
8. Maternal health conditions like Diabetes and HT are important factors in hypoglycemia but no mention in the baseline Characteristics table 1.
9. Is Maternal weight/ nutrition like BMI and socio-economic status important factor in Hypoglycemia and if so was it assessed?
10. Sick children are more likely to get hypoglycemia . Were these children all well with no fever/ infection that may affect blood sugar? There was no mention of physical characteristics of your study population like the vitals e.g. Temp. RR etc.
11. There is no mention or show of the total number of children with varying levels of RBS : For example a bar graph showing% RBS -2.6 and RBS equal to and above 2.6mmol, infact include a % of children with RBS < 2.2 mmol. This is important because of the conflicting description of Hypo in new borns.
12. I would like to see the 2x2 columns of the children who had hypo and those who didn’t have hypo by the varying characteristics. Also add the P-values of the OR
13. Was high prevalence of Hypo due to your definition of hypo. U need to clearly discuss this? Were these children at high risk of illness and other factors associated with Hypo? What was prevalence of HIV among mothers in these population? Was this group of children delivered in these hospitals likely to be the complicated deliveries and thus delay in feeds coz of complications???
14. You compare your results with Mukunya etal yet his study was among full-term babies and not preterm and thus can’t be comparable. Also, Mukunya was community study thus the children more likely stabler than those in hospital?
15. If indeed it’s the physiological mechanism of low glycogen stores that predisposes these babies to HYPO then children in the developed setting should also be getting the same effect with high frequency of Hypoglycemia like in our setting? What could explain the differences in the two settings?
16. Does the temperature also affect the blood sugar of the children. What's the prevalence of hypothermia in your setting visavis those settings with low reported Hypoglycemia?
17. I did not see any institutional factors that were investigated in this study yet the authors keep discussing / mentioning them. Please highlight some?
18. Would you expect that a cross sectional study in a few hospitals with a few investigated factors with some confounders would be used to change policy? Was this a representative population or it was a highly selective group?
19. Clearly state your Limitations in this study that may have affected your outcome?
20. Please make your recommendations more succinct.. The implications of your study should be clearly stated .
