# Supplementary material for: Asymptomatic hypoglycemia among preterm newborns: A cross-sectional analysis
Source: PLoS One. 2024 Apr 30;19(4):e0301803. doi: 10.1371/journal.pone.0301803 (PMC11060517; doi:10.1371/journal.pone.0301803)
Supplement: Attachment — Submitted filename: Reviewers responses-PLoS ONE-D-23-33081R1.doc [file pone.0301803.s004.doc]

| **Editors’/Reviewers’ comments-advice** | **Specific final change made by authors** | **Final status (page number)** |
| --- | --- | --- |
| 1. **Editors’ advice:**   Please ensure you include your title page in the main document | Title page is part of the manuscript (main) document now. | Changed as requested (pp. 1) |
| 2. **Reviewer’s advice:**   Neonatal hypoglycemia change to ‘hypoglycemia in preterm newborns’ | Changed to read as hypoglycemia in preterm newborns. | Changed as requested (pp. 1) |
| 3. **Reviewer’s advice:**   Keywords – Dar es Salaam, replace it with the word preterm. | Changed to preterm | Changed as requested (pp. 3) |
| **Reviewer’s advice:**  Abstract section:    4. “There is a growing body of evidence that links the causes of early neonatal mortality to neonatal hypoglycemia in Tanzania.”  Replace it with another statement, this is a well-established fact globally.  5. “Written informed consent was sought from each mother prior to inclusion of their babies into the study” delete  6. “Breastfeeding within 1^st^ hour post-delivery was the only factor significantly associated with neonatal hypoglycemia (O.R.: 0.123, 95% C.I.: 0.052 – 0.287)”. Kindly rephrase to avoid ambiguity. Your odds ratio | Replaced with a totally new sentence of  “Preterm newborns are the group with the highest risk of clinically significant hypoglycemia”.  Deleted.  Changed to  “Breastfeeding within 1st hour post-delivery was a statistically significant factor against glycemic levels associated with hypoglycemia (OR; 0.123, 95%-CI; 0.052-0.287) in a fitted multivariable logistic regression model” | Changed as requested (pp. 3)  Deleted as requested (pp. 3)  Changed to reflect the appropriate statistical interpretation (refer to results section pp. 3) |

| **Editors’/Reviewers’ comments-advice** | **Specific final change made by authors** | **Final status (page number)** |
| --- | --- | --- |
| **Reviewer’s comments**  Abstract section (cont…)  7. “Nearly half of all asymptomatic newborns were in hypoglycemic range.” You cannot conclude with findings not in the results section, this is appearing out of nowhere.  8. “Preterm neonates maybe the risky group worth considering for continuous glycemic screening at hospital protocols”. Your abstract failed to support this statement and thus, you cannot make this recommendation. | Changed to read as  “About half of all preterm newborns studied had glycemic values in a limit associated with hypoglycemia in newborns”.  Reason:  Definition of the term ‘median’ as a statistical measure of central tendency. The median random glycaemia among study participants was 2.6 mmol/L (see table 1 in results section pp. 7).  Deleted | Changed accordingly with retention of the glycemic value of 2.6 mmol/L  Deleted |

| **Editors’/Reviewers’ comments-advice** | **Specific final change made by authors** | **Final status (page number)** |
| --- | --- | --- |
| **Reviewer’s comments**  Introduction section:  9. “They consider their argument on possible evolutionary basis for its occurrence within the first 24-hours of extra-uterine life. They justified their thinking on the nosology to be based on symptoms and signs (clinical aspects),” replace the word “they” and avoid third person pronoun in a scientific writing.  10. “Some studies have shown perinatal hypoxia, small for gestational age and maternal hypertension to be factors associated with hypoglycemia in preterm newborns (4, 5) However, the exact mechanisms behind the observations are yet to be identified by neonatologists/endocrinologists to date. Yet still, it is not clear whether the suggested risks are generalized or specific to a certain neonatal cohort alone”.  11. “This study gives findings from a cross-sectional sample of preterm neonates who presented with asymptomatic hypoglycemia in typical neonatal set ups” Delete | Replaced with words  “Scholars antagonising the concept, base their argument primarily on possible evolutionary advantages to the phenomenon. Those scholars justified their thinking on the nosology, based on symptoms and signs (clinical aspects), since for that particular condition, quite often clinicians cannot differentiate physiology from possible pathology using figures alone”.  Moved accordingly  Deleted accordingly | Changed as per reviewer’s suggestions.  Changed as per reviewer’s suggestions.  (see pp. 4)  Deleted as per reviewer’s recommendations |

| **Editors’/Reviewers’ comments-advice** | **Specific final change made by authors** | **Final status (page number)** |
| --- | --- | --- |
| **Reviewer’s advice**:  Methods section:  12. “Otherwise, Tanzania is a nation state situated on the East African coast. It is a former German colony, that accidentally fell under British protectorate after the World War II, up to her political independence. Most of her existing infrastructure in health, especially the study settings are post-independence initiatives on health. Tanzania is among the fastest growing nation states in Africa. She is rich in demographic sects, as evidenced by different population sub-structure ranging from Bantus to Afro-Asiatic population groups, especially in Dar es Salaam” Delete, this is a manuscript and not a dissertation or thesis  13. Thus, the study population was considered to be representative of African demographic sects for all practical and clinical purposes. This is incorrect, kindly delete  14. What was the definition of neonatal hypoglycemia in preterm? | Deleted  Deleted  Neonatal hypoglycemia was defined as a random blood glucose level ≤ 2.6 mmol/L whether or not they presented with symptoms and/or signs consistent with clinically defined hypoglycemia. | Deleted as per reviewer’s advice  (see pp. 5)  Deleted as per reviewer’s recommendations (see pp. 5)    Unchanged |

| **Editors’/Reviewers’ comments-advice** | **Specific final change made by authors** | **Final status (page number)** |
| --- | --- | --- |
| **Reviewer’s advice**:  Methods section (cont…):  15. What was the definition of preterm? How did you assess prematurity?? Using LNMP or Ballard score??  16. Did you include children who had received IV dextrose infusion If so, how many hours since the infusion?  17. Are the characteristics of the children admitted at these three hospitals the same?  18. “Data was triple entered under” who do you mean by triple entered? Why? | Prematurity was assessed using Ballard score  All children who received IV dextrose infusions were excluded from the study.  For all practical and clinical purposes – yes.  Besides, the study settings (all three hospitals) are for all practical purposes considered to be the same as they are in the same level in the Tanzania’s referral system (i.e. regional referral hospitals). Furthermore, they serve as public facilities with more or less the same population in terms of demographics, socio-economic stata, etc  Data were entered thrice during data entry process by three separate data clerks.  **Reason:** To avoid possibilities for data collection and/or data entry errors to the maximum during data collection/storage stage. Multiple data clerks brought data to the entry sites, and there were therefore potential risks for erroneous data inclusion/deletion/misrepresentation. | Unchanged  Unchanged  Unchanged  Unchanged |

| **Editors’/Reviewers’ comments-advice** | **Specific final change made by authors** | **Final status (page number)** |
| --- | --- | --- |
| **Reviewer’s advice**:  Methods section (cont…):  19. “(hypoglycemia – coded as 1- if random glucose value was ≥ 2.6 mmol/L and 0 – if random glycaemia was < 2.6 mmol/L).” kindly cross-check, your desired outcome is hypoglycemia and should have been coded 1.  Results section  20. “TABLE 2: multivariate analysis”. It should be bivariate or multivariable binary logistic regression. No mention of how many has hypoglycemia | Agreed. There was a typo during typing as in reality what was done included considering outcome of interest (coded as 1) whenever random glycaemia was ≤ 2.6 mmol/L and anything else as normoglycemia (coded as 0). The sentence has been rephrased to reflect the correct meaning of what was done during exploratory data analysis and even final data analysis.  Agreed and changed accordingly | Changed accordingly  (refer pp. 6)  Changed in accordance to reviewer’s recommendations. (see pp. 7) |

| **Editors’/Reviewers’ comments-advice** | **Specific final change made by authors** | **Final status (page number)** |
| --- | --- | --- |
| **Reviewer’s advice**:  Discussion section:  21. “In this study, we found almost half of all preterm babies to have been in quantitative hypoglycemic range”. This is not mentioned in your results. | Kindly refer to table 1 in the results section under median values of neonatal glucose levels (in mmol/L). Median was the measure of central tendency used during analysis as data was found to be skewed. Besides, the median is statistically defined as the 50^th^ percentile of the values of an item/variable under study. the median value of random glycemia for the studied newborns was 2.6 mmol/L. Standard quantitative cut-off point for neonatal hypoglycemia is 2.6 mmol/L. | Unchanged |

| **Editors’/Reviewers’ comments-advice** | **Specific final change made by authors** | **Final status (page number)** |
| --- | --- | --- |
| **Reviewer’s advice**:  Discussion section:  22. ”The current findings are comparable to others in similar settings (7-10) For instance, Sultan and his colleagues found out 73% of all babies born prematurely in SouthEastern Tanzania to had been in hypoglycemic range” This statement contradict your earlier assertion that no study has been done in Tanzania (At present, there is no evidence from available/retrievable published databases of any findings on factors associated with neonatal hypoglycemia among preterm babies in Tanzania). | Yes, even though Sultan’s study was done in Tanzania, it referred to findings that were derived from studies performed using a cohort of all neonates and not specific to preterm babies. Besides, the study is almost two decades ago, and hence newer data were still unavailable at present. To remove ambiguity and controversies, the sentence has been rephrased to | Rephrased in order to account for reviewer’s recommendations. |
